# Calcium Signaling in the Ventricular Myocardium of the Goto-Kakizaki Type 2 Diabetic Rat

**DOI:** 10.1155/2018/2974304

**Published:** 2018-04-10

**Authors:** L. Al Kury, M. Smail, M. A. Qureshi, V. Sydorenko, A. Shmygol, M. Oz, J. Singh, F. C. Howarth

**Affiliations:** ^1^College of Natural and Health Sciences, Zayed University, Abu Dhabi, UAE; ^2^Department of Physiology, College of Medicine & Health Sciences, UAE University, Al Ain, UAE; ^3^Department of Cellular Membranology, Bogomoletz Institute of Physiology, Kiev, Ukraine; ^4^Department of Basic Medical Sciences, College of Medicine, Qatar University, Doha, Qatar; ^5^School of Forensic & Applied Sciences, University of Central Lancashire, Preston, UK

## Abstract

The association between diabetes mellitus (DM) and high mortality linked to cardiovascular disease (CVD) is a major concern worldwide. Clinical and preclinical studies have demonstrated a variety of diastolic and systolic dysfunctions in patients with type 2 diabetes mellitus (T2DM) with the severity of abnormalities depending on the patients' age and duration of diabetes. The cellular basis of hemodynamic dysfunction in a type 2 diabetic heart is still not well understood. The aim of this review is to evaluate our current understanding of contractile dysfunction and disturbances of Ca^2+^ transport in the Goto-Kakizaki (GK) diabetic rat heart. The GK rat is a widely used nonobese, nonhypertensive genetic model of T2DM which is characterized by insulin resistance, elevated blood glucose, alterations in blood lipid profile, and cardiac dysfunction.

## 1. Use of the Goto-Kakizaki Diabetic Rat

Diabetes mellitus (DM) is a metabolic disease characterized by abnormal glucose homeostasis and defects in insulin metabolism. Cardiovascular disease (CVD) is the leading cause of death in the diabetic population. However, the molecular mechanisms underlying diabetic cardiomyopathy remain unclear.

Animal models are increasingly being used to elucidate the mechanisms underlying diabetic cardiomyopathy in both type 1 and type 2 diabetes. One of the most widely used animal models of type 2 diabetes mellitus (T2DM) is the Goto-Kakizaki (GK) rat. The GK rat is a polygenic nonobese model of T2DM. This model is generated by selective inbreeding of mildly glucose-intolerant Wistar rats over many generations [1]. At least 17 genes associated with metabolism, signal transduction, receptors, and secreted factors are involved in the pathogenesis of diabetes in the GK rat [2]. The general characteristics of the GK rat include fasting hyperglycemia, impaired insulin secretion in response to glucose both *in vivo* and in isolated pancreata, raised glycosylated hemoglobin, hepatic and peripheral insulin resistance, altered heart and body weight, and a variety of late complications, including cardiomyopathy, nephropathy, and neuropathy [1, 3–11]. In contrast to many other non-insulin-dependent rodent models, GK rats are non-obese [1, 12].

Three genetic loci are responsible for coding and transferring diabetic pathology to the fetus, and these include genes that are responsible for a reduction in *β*-cell mass and reduced insulin secretion [12]. During the prediabetic period (first three weeks after birth), animals have reduced body weight and do not show hyperglycemia. After weaning, many changes occur which include hyperglycemia, impaired glucose-induced insulin secretion (due to defective prenatal *β*-cell proliferation and reduction in *β*-cell mass), reduced insulin sensitivity in the liver, and moderate insulin resistance in peripheral tissues [12, 13].

Persistent hyperglycemia over time provokes pancreatic islet inflammation, oxidative stress, fibrosis, and finally *β*-cell dysfunction. In fact, the pancreatic islets of adult GK rats show decreased *β*-cell number and insulin content as compared to their age-matched control animals [12].

GK rats have been considered as one of the best nonobese type 2 diabetic animal models. GK rats exhibit valuable characteristics that are more or less common and functionally present in human diabetic patients. This animal model is considered appropriate to examine various pathologic mechanisms of T2DM [12, 14]. As mentioned earlier, reduced *β*-cell mass and reduced *β*-cell function are key characteristics found in this animal model [15]. Therefore, it is clear that GK rats form an important resource in preclinical T2DM research [16] in order to study the role of *β*-cell compensation in the pathogenesis of T2DM.

An earlier study has shown that GK islet fibrosis is accompanied by marked inflammation which is a characteristic that has been reported in islets of type 2 diabetic patients [17]. Other changes that are common between GK rats and human diabetic patients include decreased activity of glucose transporter (GLUT-2), glycerol-3-phosphate dehydrogenase (GPDH), and glucokinase and changes in the lipid profile [12].

As in humans, GK rats also develop renal lesions, structural changes in peripheral nerves, and retinal damage [13]. For example, in adult GK rats, significant morphological alterations in kidneys occur in response to chronic hyperglycemia which are similar to that in human diabetic patients [18, 19]. These morphological changes in kidneys include glomerulosclerosis, proliferation of mesangial cells, atrophy of basement membrane, and tubulointerstitial fibrosis [20].

## 2. Other Animal Models of Type 2 Diabetes

T2DM is characterized by insulin resistance and the inability of the *β*-cell to sufficiently compensate, which leads to hyperglycemia [21]. In addition, T2DM is closely associated with obesity which is one of the main pathological causes of insulin resistance [15, 22]. Many animal models are therefore obese as a result of naturally occurring mutations or genetic manipulation and are useful in understanding obesity-induced insulin resistance and its effects. These are divided into monogenic models, polygenic models, and diet-induced models [23]. The general characteristics for these obese models are insulin resistance and impaired glucose tolerance. In other words, these models lack sufficient insulin secretion required to compensate for the insulin resistance as part of the obesity (obesity-induced hyperglycemia) [13, 23].

Lep^ob/ob^ mice, Lepr^db/db^ mice, and Zucker diabetic fatty rats are the most commonly used models of monogenic obesity. They have a disrupted leptin signaling pathway, leading to hyperphagia and obesity [13]. Polygenetic animal models, however, provide more accurate models of the human condition [15]. These include KK-A^Y^ mice, New Zealand obese (NZO) mice, TallHo/Jng mice, and Otsuka Long Evans Tokushima Fat (OLETF) rat. Obesity can also be induced by feeding the rodent a high-fat diet (diet-induced models). The weight gain in these animals is associated with insulin resistance and abnormal glucose metabolism [12, 13, 23].

In contrast to the animal models mentioned above, the GK rat is a non-obese animal model of T2DM. It is characterized by reduced *β*-cell mass and/or *β*-cell function [24]. The GK rat is glucose intolerant and displays defective glucose-induced insulin secretion. Furthermore, the development of insulin resistance does not seem to be the main initiator of hyperglycemia. Instead, the defective glucose metabolism is regarded to be due to reduced *β*-cell mass [25] and/or function [26]. Adult GK rats show a 60% decrease in their total pancreatic *β*-cell mass. Blood glucose is elevated only after the first 3-4 weeks of animal's age, and blood glucose rises significantly after a glucose challenge [13, 27]. The GK model is characterized by early hyperglycemia, hyperinsulinemia, and insulin resistance, [1, 12]. Other examples of non-obese animal models of T2DM are the C57BL/6 (Akita) mutant mouse, the Cohen diabetic rats, and the spontaneously diabetic Torri (SDT) rats [13].

## 3. Blood Chemistry in the Goto-Kakizaki Diabetic Rat

Blood insulin, glucose, and lipid profiles in the GK rats compared to controls are summarized in Tables 1, 2, and 3, respectively. Blood insulin is either unaltered [28–34] or increased [29, 34, 35] in the GK rats (Table 1). Fasting blood glucose and nonfasting blood glucose are slightly increased [10, 11, 28–48] and urine glucose is increased [30] in the GK rat. Following a glucose challenge, in the fasted state, blood glucose is significantly elevated at 30, 60, and 120 min [29, 37–40, 44, 46, 48–50] in the GK rat indicating end organ resistance to the action of insulin (Table 2). Blood cholesterol is increased [29, 35, 43, 44] whilst high-density lipoprotein cholesterol may be either unaltered [31] or increased [44] and low-density lipoprotein cholesterol is unaltered [31, 44] in the GK rat compared to controls. Blood free fatty acids are either unaltered [11, 31] or increased [38, 45] in the GK rats compared to controls. Triglycerides are either increased [38, 43–45] or unaltered [2, 30, 45] in the GK rats compared to controls (Table 3). Part of the variability in blood chemistry may be attributed to the age of the animals and dietary regime. In summary, the GK rat displays hyperglycemia, insulin resistance, and disturbances in lipid profile.

## 4. Body and Heart Weight in the Goto-Kakizaki Diabetic Rat

Body weight and heart weight measures in GK rats compared to controls are summarized in Tables 4 and 5, respectively. Body weight is either unaltered [31, 34, 36, 39–41, 46, 50], decreased [2, 10, 11, 28–30, 32, 35, 38, 42–46], or increased [34, 47, 48] in the GK rat (Table 4). Heart weight is generally increased [29, 40, 41, 48, 49] but may also be decreased [10, 43] or unaltered [11, 39]; left ventricular weight is either decreased [43, 45] or increased [32]; left ventricular thickness is increased [40] or unaltered [36]; right ventricular weight is either unaltered [45] or decreased [45] in GK rats compared to controls. Heart-weight-to-body-weight ratio is increased [10, 11, 29, 30, 32, 33, 36, 40, 50] but may also be unaltered [31, 41, 48]; heart-weight-to-femur-length ratio is increased [44]; left-ventricle-to-body-weight ratio is increased [36, 43, 45, 51]; right-ventricle-to-body-weight ratio is unaltered [45]; biventricular-weight-to-body-weight and biventricular-weight-to-tibial-length ratios are increased [28, 45] (Table 5). In summary, the various heart to body ratio measures and the structural changes observed in the heart of this nonobese, nonhypertensive animal model provide evidence for regional cardiac hypertrophy.

Earlier studies have reported that chronic mild hyperglycemia produces molecular and structural correlates of hypertrophic myopathy in GK rats [40]. Several mechanisms whereby hyperglycemia may induce left ventricle remodeling have been proposed. One of these mechanisms is the increased activity of profibrotic and prohypertrophic cytokine transforming growth factor-*β*1 (TGF-*β*1) in the ventricular tissue [52]. TGF-*β*1 reproduces most of the hallmarks seen in structural remodeling. Specifically, TGF-*β*1 induces expression levels of extracellular matrix (ECM) constituents by cardiac fibroblasts (i.e., fibrillar collagen, fibronectin, and proteoglycans), self-amplifies its own expression in both cardiac myocytes and fibroblast [53, 54], and stimulates the proliferation of fibroblasts and their phenotypic conversion to myofibroblasts [55, 56]. D'Souza et al. have shown that the increased activity of TGF-*β*1 and phosphorylation of protein kinase B (PKB)/Akt and its downstream effectors mediate the hypertrophic effects of TGF-*β*1 in the prediabetic GK left ventricle [36]. The hypertrophic events were also sustained in the aging GK myocardium [40]. Earlier studies have suggested that enhanced activity of myocardial Na^+^/H^+^ exchanger plays a role in the molecular mechanisms involved in cardiac hypertrophy. It is likely that the activation of the Akt pathway mediates the hypertrophic effect of myocardial Na^+^/H^+^ exchanger in the GK rat model of T2DM [28]. Interestingly, several studies have shown that female rat hearts are more hypertrophied than male hearts [10, 32, 57].

## 5. *In Vivo* Hemodynamic Function in the Goto-Kakizaki Rat Heart

*In vivo* hemodynamic function and related measures in GK rats compared to controls are summarized in Table 6. Heart rate is either unaltered [28, 30–33, 37, 45, 58] or reduced [2, 34, 46] in the GK rat. Systolic blood pressure is unaltered [28, 30, 31, 33, 58] or increased [32, 34, 37, 58]; whilst diastolic blood pressure is increased [30, 34], mean arterial pressure is unaltered [35], increased [37], or reduced [30] in GK rat. Rate for pressure development (+dP/dt) and decline (–dP/dt) in left ventricle is unaltered [30, 45] in the GK rat. Ejection fraction is reduced [28, 51], increased [44], or unaltered [30, 33]; fractional shortening is reduced [32, 51] or unaltered [2, 33, 45]; cardiac output is unaltered [33] or decreased [51] in the GK rat. Coronary blood flow is increased [29] or reduced [2] in GK rats compared to controls. In summary, the GK rat heart may display a variety of abnormal hemodynamic characteristics including alterations in heart rate, blood pressure, blood pumping capability, and altered coronary blood flow.

## 6. Hemodynamic Function in the Isolated Perfused Goto-Kakizaki Rat Heart

Heart rate in the isolated perfused heart is lower in comparison to the heart rate *in vivo* in GK and control hearts (Table 7). Isolated perfused heart rate is unaltered [10, 11, 31, 50] in GK rats. Left ventricle +dP/dt and –dP/dt are either unaltered [10, 31, 59] or reduced [51] in the GK rat. Coronary flow is either reduced [11, 31] or unaltered [10] in GK rats compared to controls. Collectively, the GK rat heart displays a variety of abnormal hemodynamic characteristics, including altered rate of development and relaxation of ventricular contraction and altered coronary flow compared to controls.

## 7. Contraction in Ventricular Myocytes from the Goto-Kakizaki Rat Heart

Characteristics of shortening in myocytes from GK rats compared to controls are shown in Table 8. Myocyte diameter, surface area, cross-sectional area, and cell capacitance were increased [28, 30, 33, 36, 40, 51], and resting cell length may be unaltered [10, 39, 41, 50] or increased [47] in myocytes from the GK rat. In electrically stimulated myocytes, the time-to-peak (TPK) shortening was prolonged [39, 41, 47] or unaltered [48, 50] and the time-to-half (THALF) relaxation of shortening may be unaltered [41, 47, 48] or shortened [50] or lengthened [39] in myocytes from the GK rat. Amplitude of shortening may be unaltered [10, 41, 48, 50] or increased [39] in myocytes from the GK rat. In summary, ventricular myocytes from the GK rat heart tend to be larger in size and have prolonged time course and generally similar amplitude of contraction compared to myocytes from the control heart.

During the process of excitation-contraction coupling (ECC), the arrival of an action potential causes depolarization of the cardiac myocyte plasma membrane. This depolarization opens voltage-gated L-type Ca^2+^ channels in the plasma membrane. The entry of small amounts of Ca^2+^ through these channels triggers a large release of Ca^2+^ from the sarcoplasmic reticulum (SR) via activation of the ryanodine receptor (RyR), by the process termed calcium-induced calcium release (CICR). The transient rise in intracellular Ca^2+^ (Ca^2+^ transient) results in the binding of Ca^2+^ to troponin C which initiates and regulates the process of cardiac muscle cell contraction. During the process of relaxation, Ca^2+^ is pumped back into the SR via the SR Ca^2+^-ATPase (SERCA2) and extruded from the cell, primarily via the Na^+^/Ca^2+^ exchanger (NCX) [60, 61]. Changes in the kinetics of shortening observed in myocytes of GK rats may be attributed, at least in part, to alternations in ventricular myocardial stiffness. Earlier studies have demonstrated increased collagen deposition and increased ventricular stiffness in different experimental models of T2DM, which in turn were associated with altered kinetics of myocardial contraction [62, 63]. The observed disturbance in myocyte shortening may also be attributed to the alternation in the profile of expression of mRNA encoding various proteins involved in excitation-contraction coupling [48].

## 8. Intracellular Ca^2+^ in Ventricular Myocytes from the Goto-Kakizaki Rat Heart

Characteristics of intracellular Ca^2+^ in myocytes from GK rats compared to controls are shown in Table 9. Resting intracellular Ca^2+^ is unaltered [10, 41, 47, 48] or increased [28]; TPK Ca^2+^ transient is unaltered [39, 41, 48, 50] or prolonged [47]; THALF decay of the Ca^2+^ transient is unaltered [39, 47, 48, 50] or shortened [41]; and the amplitude of the Ca^2+^ transient is unaltered [10, 41, 48], increased [47, 50], or decreased [39] in myocytes from the GK rat. In whole-cell patch clamp experiments, the amplitude, inactivation, and restitution of L-type Ca^2+^ current are unaltered [48] in myocytes from GK rats compared to controls.

Since intracellular Ca^2+^ in cardiac cells is maintained by Ca^2+^ influx (through L-type Ca^2+^ channels; the primary trigger for SR Ca^2+^ release) and efflux (through NCX; the major pathway for Ca^2+^ efflux from the cell) [64], as well as Ca^2+^ release (via the ryanodine receptors) and uptake by both SR (through SERCA2) and mitochondria, it is possible that the observed differences in these results may be attributed to differential changes in Ca^2+^ transport activities in these organelles. Furthermore, the observed alterations in intracellular Ca^2+^ may also be due to differences in the stage and severity of diabetes [65, 66].

It is well known that alterations in SR Ca^2+^ uptake and release mechanisms would impair cardiac cell function. Several studies have reported changes in cardiac SR Ca^2+^ transport during the development of chronic diabetes [67–71]. For example, Ganguly et al. reported that a decrease in Ca^2+^ uptake activity by SR was associated with a decrease in SERCA2a activity [68]. Furthermore, Golfman et al. showed that SR ATP-dependent Ca^2+^ uptake activity was markedly decreased in the diabetic rat heart [72]. Yu et al. reported a reduction in both SR Ca^2+^ content and ryanodine binding sites in diabetic hearts, indicating that the SR functions of storage and release of Ca^2+^ were depressed [73]. It should be noted that prolonged depression of the SR Ca^2+^ uptake activity in chronic diabetes may contribute to the occurrence of intracellular Ca^2+^ overload [65].

In our recently published data, L-type Ca^2+^ current and Ca^2+^ transients were simultaneously measured in endocardial (ENDO) and epicardial (EPI) myocytes from the left ventricle of GK rats [74]. Consistent with previous findings [48], the amplitude of L-type Ca^2+^ current, over a wide range of test potentials, was unaltered in ENDO and EPI myocytes from the left ventricle of GK rat. However, the amplitude of the Ca^2+^ transients was reduced and by similar extents, in ENDO and EPI myocytes from the GK rat heart. The THALF decay of the Ca^2+^ transients was reduced in EPI and ENDO myocytes from GK rats compared to controls. Interestingly, while a reduction in the amplitude of L-type Ca^+^ current has been reported in earlier studies on a diabetic heart [75, 76], it does not necessarily explain the reduced Ca^2+^ transients. This is because many reports show no change in L-type Ca^2+^ current despite the reduction in both contractions and Ca^2+^ transients [48, 74, 77–79]. Instead, reduction of Ca^2+^ transients and the consequent contractile dysfunction may be due to depletion of SR Ca^2+^, which may result from RYR-dependent Ca^2+^ leak, an increased Ca^2+^ extrusion through NCX, or a reduced function of SERCA [61, 80]. Further experiments will be required to investigate the role of SR in Ca^2+^ transport in myocytes from the GK rat. Sheikh et al. [81] demonstrated that cardiac endothelial cells from diabetic rats treated with NCX inhibitor have higher intracellular Ca^2+^ transient peaks as compared to controls. This finding supports the idea that altered activity of sarcolemmal NCX during Ca^2+^ efflux contributes to the decrease in Ca^2+^ transient-observed GK myocytes. Previous experiments in ventricular myocytes from the streptozotocin-induced diabetic rats have reported reduced caffeine-evoked Ca^2+^ transients [82–91], SERCA2 activity, and Ca^2+^ uptake [83, 88, 92–94] and decreased SR Ca^2+^ channel (ryanodine receptor) activity [87, 95] suggesting decreased SR Ca^2+^ content, Ca^2+^ uptake, and Ca^2+^ release mechanisms in ventricular myocytes from the streptozotocin-induced diabetic rat.

Under pathological conditions, such as chronic diabetes, the mitochondria are able to accumulate large amounts of Ca^2+^, which serves as a protective mechanism during cardiac dysfunction and intracellular Ca^2+^ overload. Therefore, altered mitochondrial uptake of Ca^2+^ during diabetes may contribute to the reported decreased Ca^2+^ transients. Although the mitochondria contribute to Ca^2+^ signaling, their exact role in diabetic cardiomyopathy remains to be investigated.

Recent investigations, using animal models, suggest that mitochondrial dysfunction may also play a critical role in the pathogenesis of diabetic cardiomyopathy [65, 71]. Potential mechanisms that contribute to mitochondrial impairment in diabetes include altered energy metabolism [96–99] oxidative stress [100–102], altered mitochondrial dynamics and biogenesis [103, 104], cell death [105, 106], and impaired mitochondrial Ca^2+^ handling [107, 108].

It should be noted that the main function of the mitochondria in the heart is to produce energy in the form of ATP, which is required for cardiac contractile activity. However, mitochondria are known to serve as Ca^2+^ sinks in the cell by acting as a local buffering system, removing Ca^2+^ and modulating cytosolic Ca^2+^concentrations [65, 109]. In addition to controlling their intraorganelle Ca^2+^ concentration, mitochondria dynamically interact with the cytosol and intracellular Ca^2+^ handling machineries to shape the cellular Ca^2+^ signaling network [65]. Recent evidence suggests that there is a dynamic exchange of Ca^2+^ between the mitochondria and the cytosol and that mitochondrial Ca^2+^ uptake increases mitochondrial ATP production [110]. Therefore, mitochondria can play an important role in preventing and/or delaying the occurrence of intracellular Ca^2+^ overload in cardiomyocytes under different pathological conditions. For example, during the development of cardiac dysfunction and intracellular Ca^2+^ overload in chronic diabetes, mitochondria are believed to continue accumulating Ca^2+^, thereby serving as a protective mechanism [65, 71]. However, when the intramitochondrial Ca^2+^ concentration exceeds its buffering capacity, irreversible swelling occurs leading to mitochondrial dysfunction. As a result, energy production as well as energy stores are depleted. Collectively, these defects may contribute to the development of cardiac dysfunction in diabetic cardiomyopathy [109].

Evidence of deficits in mitochondrial Ca^2+^ handling has been demonstrated in animal models of both type 1 and type 2 diabetes. For example, in streptozotocin- (STZ-) induced diabetic rats, hyperglycemia was associated with lower rates of mitochondrial Ca^2+^ uptake [107]. This reduction can be explained by the increased opening of the mitochondrial permeability transition pore (MPTP), resulting in the release of accumulated Ca^2+^. In STZ-induced diabetic rats, Oliveira et al. observed that Ca^2+^ uptake was similar in control versus diabetic hearts; however, mitochondria in diabetic hearts were unable to retain the accumulated Ca^2+^. This effect was not observed in the presence of cyclosporin, an MPTP inhibitor [108]. In type 2 diabetic ob/ob mice, reduced intracellular Ca^2+^ release upon electrical stimulation, slowed intracellular Ca^2+^ decay rate, and impaired mitochondrial Ca^2+^ handling were observed [111, 112]. Similarly, Belke et al. observed a reduction in Ca^2+^ levels and a reduction in the rate of Ca^2+^ decay in isolated cardiomyocytes from *db/db* animals, suggesting impaired mitochondrial Ca^2+^ uptake [113]. Taken together, these studies support the notion that mitochondrial Ca^2+^ handling is impaired in diabetic myocardium, resulting in compromised energy metabolism and thus reduced contractility.

## 9. Conclusion

**Figure 1:**
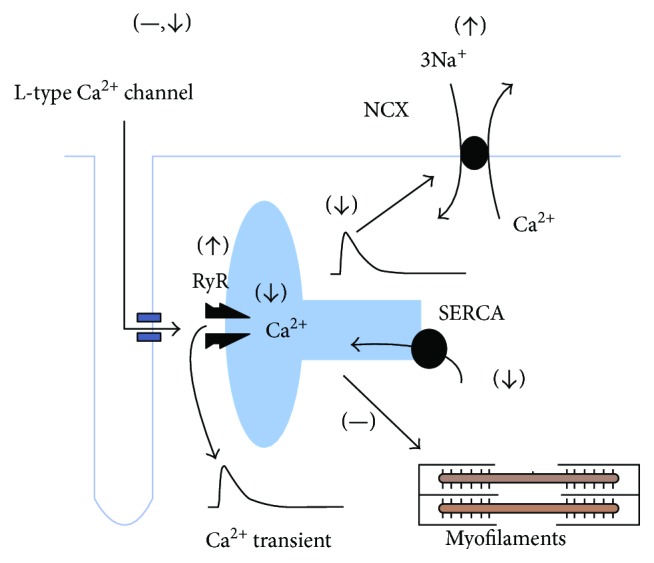
Schematic diagram showing the summary of some of the proposed mechanisms involved in the alterations in Ca^2+^ signaling in cardiac myocyte from the GK diabetic heart. (1) No change/or decrease in L-type Ca^2+^ channel activity, (2) increase in Na^+^/Ca^2+^ exchange current, (3) decrease in SR Ca^2+^ content, (4) decrease in SR Ca^2+^ uptake, and (5) increase in Ca^2+^ release through RYR. SR: sarcoplasmic reticulum; RYR: ryanodine receptor; SERCA: sarcoplasmic reticulum Ca^2+^-ATPase; NCX: Na^+^/Ca^2+^ exchanger; —: no effect; ↑: increased activity; ↓: decreased activity (adapted from Eisner, 2013).

Although diabetic cardiomyopathy is a frequent and important complication of DM, its physiological bases are still not completely understood. The GK type 2 diabetic heart displays a variety of abnormal hemodynamic characteristics *in vivo* and in the isolated perfused heart. Hyperglycemia is usually associated with alterations in heart rate, blood pressure, blood pumping capability, and/or coronary blood flow. Contractile function, in terms of amplitude and kinetics of shortening, is frequently disturbed in the GK type 2 diabetic heart. Several mechanisms may contribute to cardiac dysfunction including mitochondrial dysfunction, myocardial fibrosis, hypertrophy, and apoptosis. Many studies show no change in L-type Ca^2+^ current despite the reduction in both contractions and Ca^2+^ transient. Instead, reduction of Ca^2+^ transients and the consequent contractile dysfunction may be attributed to both depletion of SR Ca^2+^, which may result from RyR-dependent Ca^2+^ leak, an increased Ca^2+^ extrusion through NCX, or a reduced function of SERCA (Figure 1). Understanding the molecular mechanism(s) of altered Ca^2+^ signaling will provide opportunities for the development of new treatments to improve heart function in T2DM patients.

## Figures and Tables

**Table 1:** Blood insulin in the GK rat.

Parameter	Age	Control versus GK	Reference
INS	5, 15, and 22 w	79.2 versus 77.4 [5], 151.4 versus 165.2^∗^ [15], and 171.5 versus 234.1^∗^ [22] (pmol/l)	[34]
	7, 11, and 15 w	Increased at 7^∗^ and 11 w^∗^, unaltered at 15 w	[29]
	14–16 w	150 versus 176 pmol/l NSD	[28]
	16 w	1.60 versus 2.11^∗^ (*μ*g/ml)	[35]
	16 w	6.3 versus 5.3 mU/l NSD	[30]
	18 w	4.9 versus 2.1 ng/ml NSD	[31]
	20 w	4.1 versus 2.6 ng/ml NSD	[32]
	20 w	1.7 versus 2.2 pg/ml NSD	[33]
	5, 15, and 22 w	79.2 versus 77.4 [5], 151.4 versus 165.2 [15], and 171.5 versus 234.1^∗^ [22] (pmol/l)	[34]
	24 w	14.5 versus 12.32 *μ*g/ml NSD	[2]
	18 and 30 w	132 versus 87^∗^ [18] and 240 versus 85^∗^ [30] (pmol/ml)	[45]

INS: insulin; NSD: no significant difference. ^∗^Significant difference.

**Table 2:** Glucose profile in the GK rat.

Parameter	Age	Control versus GK	Reference
FBG	8 w	76.2 versus 107.0^∗^ (mg/dl)	[36]
	5, 15, and 22 w	6.14 versus 7.49^∗^ [5], 7.56 versus 8.71^∗^ [15], and 5.26 versus 9.02^∗^ [22] (mmol/l)	[34]
	7, 11, and 15 w	Increased at 7^∗^, 11^∗^, and 15^∗^ (w)	[29]
	16 w	4.8 versus 8.8^∗^ (mmol/l)	[35]
	26 w	Increased^∗^	[37]
	26 w	65.8 versus 99.1^∗^ (mg/dl)	[38]
	17 m	72.1 versus 151.5^∗^ (mg/dl)	[39]
	18 m	95.2 versus 131.4^∗^ (mg/dl)	[40]
	18 m	44 versus 51 mg/dl NSD	[50]

NFBG	8–10 w	118.40 versus 166.40^∗^ (mg/dl)	[41]
	11 w	7.40 versus 9.18^∗^ (mM)	[42]
	12 w	9.02 versus 26.57^∗^ (mmol/l)	[43]
	14–16 w	9.4 versus 14.3^∗^ (mmol/l)	[28]
	16 w	8.5 versus 12.8^∗^ (mmol/l)	[30]
	18 w	6.0 versus 12.7^∗^ (mM)	[31]
	20 w	7.5 versus 17.9^∗^ (mmol/l)	[32]
	20 w	4.9 versus 8.2^∗^ (mmol/l)	[33]
	5, 15, and 22 w	6.14 versus 7.49^∗^ [5], 7.56 versus 8.71^∗^ [15], and 5.26 versus 9.02^∗^ [22] (mmol/l)	[34]
	26 w	204.42 versus 531.71^∗^ (mg/dl)	[44]
	18 and 30 w	18.7 versus 24.9^∗^ [18] and 19.2 versus 27.6^∗^ [30] (*μ*mol/ml)	[45]
	3, 6, and 15 m	49.6 versus 48.4 [3], 48.1 versus 73.3^∗^ [6], and 68.6 versus 113.3^∗^ [15] (mg/dl)	[46]
	5–8 m	11.3 versus 14.7^∗^ (mmol/l)	[10]
	9–14 m	10.3 versus 17.0^∗^ (mM)	[11]
	10 m	95.77 versus 143.06^∗^ (mg/dl)	[47]
	10-11 m	91.67 versus 161.29^∗^ (mg/dl)	[48]
	17 m	101.4 versus 188.8^∗^ (mg/dl)	[39]

UG	16 w	0.13 versus 0.73^∗^ (g/l)	[30]

OGTT	8 w	Elevated at 30^∗^, 60^∗^, and 120^∗^ (min)	[36]
	15 w	Elevated at 30^∗^, 60^∗^, and 120^∗^ (min)	[29]
	16 w	Elevated at 30^∗^ and 60^∗^ (min)	[37]
	26 w	Elevated at 15^∗^ and 60^∗^ (min)	[44]
	26 w	83.2 versus 303.4^∗^ (mg/dl) at 120 min	[38]
	10-11 m	93.93 versus 236.27^∗^ (mg/dl) at 120 min	[48]
	15 m	183.3 versus 276.9^∗^ (mg/dl) at 120 min	[46]
	17 m	148.1 versus 570.8^∗^ (mg/dl) at 120 min	[39]
	18 m	Elevated at 30^∗^, 60^∗^, 120^∗^, and 180^∗^ (min)	[40]
	18 m	153.4 versus 436.3^∗^ (mg/dl) at 180 min	[50]

OGTT	15 w	Increased^∗^ area under curve	[29]

HbA1c	25 w	3.5 versus 5.4^∗^ (%)	[38]
	5–8 m	4.0 versus 4.8^∗^ (%)	[10]

HOMA-IR	7, 11, and 15 w	Increased at 7^∗^, 11^∗^, and NSD 15 (w)	[29]

FBG: fasting blood glucose; NFBG: nonfasting blood glucose; UG: urine glucose; OGTT: oral glucose tolerance test; HbA1c: glycated hemoglobin A1c; HOMA-IR: homeostasis model assessment-estimated insulin resistance; NSD: no significant difference. ^∗^Significant difference.

**Table 3:** Lipid profile in the GK rat.

Parameter	Age	Control versus GK	Reference
CHOL	7, 11, and 15 w	Increased at 7^∗^, 11^∗^, and 15^∗^ (weeks)	[29]
	12 w	1.34 versus 2.15^∗^ (mmol/l)	[43]
	16 w	1.71 versus 1.98^∗^ (mmol/l)	[35]
	16 w	70 versus 93 mg/dl NSD	[30]
	26 w	55.57 versus 93.0^∗^ (mg/dl)	[44]

HDL CHOL	18 w	26.9 versus 29.1 mg/ml NSD	[31]
	26 w	22.0 versus 41.85^∗^ (mg/dl)	[44]

LDL CHOL	18 w	35.4 versus 39.5 mg/ml	[31]
	26 w	20.42 versus 25.34 mg/dl	[44]

FFA	18 w	0.61 versus 0.54 mM NSD	[31]
	18 and 30 w	0.30 versus 0.60^∗^ [18] and 0.41 versus 0.53^∗^ [30] (*μ*mol/ml)	[45]
	26 w	0.55 versus 1.3^∗^ (mM)	[38]
	9–14 m	0.2 versus 0.3 mM NSD	[11]

TG	12 w	0.54 versus 1.21^∗^ (mmol/l)	[43]
	16 w	1.72 versus 0.85^∗^ (mmol/l)	[35]
	16 w	67 versus 60 mg/dl NSD	[30]
	24 w	877.01 versus 1219.97 *μ*mol/l NSD	[2]
	26 w	98.2 versus 134.9^∗^ (mg/dl)	[38]
	26 w	65.14 versus 129.42^∗^ (mg/dl)	[44]
	18 and 30 w	0.74 versus 0.91 [18] and 0.93 versus 1.35^∗^ [30] (mg/ml)	[45]

CHOL: cholesterol; HDL: high-density lipoproteins; LDL: low-density lipoproteins; FFA: free fatty acids; TG: Triglycerides; NSD: no significant difference. ^∗^Significant difference.

**Table 4:** Body weight of the GK rat.

Parameter	Age	Control versus GK	Reference
BW	5, 15, and 22 w	82.0 versus 106.9^∗^ [5], 311.8 versus 315.0^∗^ [15], and 464.3 versus 417.8^∗^ [22] (g)	[34]
	8 w	325.25 versus 329.00 g NSD	[36]
	8–10 w	218.50 versus 246.40 g NSD	[41]
	11 w	402 versus 275^∗^ (g)	[42]
	12 w	432 versus 353^∗^ (g)	[43]
	15 w	Reduced^∗^	[29]
	14–16 w	376 versus 330^∗^ (g)	[28]
	16 w	481.3 versus 414.0^∗^ (g)	[35]
	16 w	450 versus 331^∗^ (g)	[30]
	18 w	376 versus 372 g NSD	[31]
	20 w	437 versus 385^∗^ (g)	[32]
	5, 15, and 22 w	82 versus 106.9^∗^ [5], 311.8 versus 315 [15], and 464.3 versus 417.8 [22] (g)	[34]
	24 w	491.67 versus 334.17^∗^ (g)	[2]
	26 w	453.8 versus 401.7^∗^ (g)	[44]
	26 w	402.3 versus 351.4^∗^ (g)	[38]
	18 and 30 w	501 versus 386^∗^ [18] and 643 versus 427^∗^ [30] (g)	[45]
	2, 7, and 10 m	205.7 versus 230.3 [2], 469.9 versus 417.5^∗^ [7], and 494.0 versus 406.3^∗^ [10] (g)	[46]
	5–8 m	559.5 versus 379.6^∗^ (g)	[10]
	9–14 m	628 versus 396^∗^ (g)	[11]
	10 m	383.31 versus 442.38^∗^ (g)	[47]
	10-11 m	400.3 versus 443.64^∗^ (g)	[48]
	17 m	436 versus 399 g NSD	[39]
	18 m	418.7 versus 413.4 g NSD	[40]
	18 m	513.4 versus 457.9 g NSD	[50]

BW: body weight; NSD: no significant difference. ^∗^Significant difference.

**Table 5:** Heart weight and other heart-related measurements in the GK rat.

Parameter	Age	Control versus GK	Reference
HW	8 w	0.807 versus 0.927^∗^ (g)	[36]
	8–10 w	0.96 versus 1.05^∗^ (g)	[41]
	12 w	1.14 versus 0.98^∗^ (g)	[43]
	15 w	Increased^∗^	[29]
	5–8 m	1700 versus 1460^∗^ (mg)	[10]
	9–14 m	2.0 versus 1.8 g NSD	[11]
	10-11 m	1.37 versus 1.60^∗^ (g)	[48]
	17 m	1.52 versus 1.50 g NSD	[39]
	18 m	1.22 versus 1.41^∗^ (g)	[40]

LVW	12 w	0.81 versus 0.68^∗^ (g)	[43]
	18 and 30 w	1.12 versus 0.86^∗^ [18] and 1.32 versus 1.03^∗^ [30] (g)	[45]
	20 w	Increased^∗^	[32]

LVT	8 w	2.98 versus 3.15 mm NSD	[36]
	18 m	3.08 versus 3.35^∗^ (mm)	[40]

RVW	18 and 30 w	0.30 versus 0.26 [18] and 0.32 versus 0.28^∗^ [30] (g)	[45]

HW/BW	8 weeks	0.248 versus 0.281^∗^ (g/100 g)	[36]
	8–10 w	4.43 versus 4.33 mg/g NSD	[41]
	15 w	Increased^∗^	[29]
	16 w	2.96 versus 3.73^∗^	[30]
	18 w	2.2 versus 2.2 NSD	[31]
	20 w	Increased^∗^	[32]
	20 w	Increased^∗^	[33]
	9–14 m	3.1 versus 4.5^∗^	[11]
	5–8 m	3.0 versus 3.8^∗^ (mg/g)	[10]
	10-11 m	3.43 versus 3.61 mg/g NSD	[48]
	18 m	0.21 versus 0.34^∗^ (g/100 g)	[40]
	18 m	3.36 versus 4.10^∗^ (mg/g)	[50]

HW/FL	26 w	0.44 versus0.49^∗^	[44]

LV/BW	8 w	1.76 versus 1.98^∗^ (mg/g)	[36]
	12 w	1.85 versus 1.95^∗^ (mg/g)	[43]
	18 and 30 w	2.16 versus 2.24^∗^ [18] and 2.06 versus 2.40^∗^ [30] (mg/kg)	[45]
	6 m	0.20 versus 0.24^∗^ (%)	[51]

RV/BW	18 and 30 (w)	0.60 versus 0.71 [18] and 0.50 versus 0.66 [30] (mg/g)	[45]

BVW/BW	14–16 w	Increased^∗^	[28]
	18 and 30 w	2.76 versus 2.94^∗^ [18] and 2.56 versus 3.06^∗^ [30] (mg/g)	[45]

BVW/TL	14–16 w	Increased^∗^	[28]

HW: heart weight; LVM: left ventricular weight; LVT: left ventricular thickness; RVW: right ventricular weight; BW: body weight; FL: femur length; BVW: biventricular weight; TL: tibial length; NSD: no significant difference. ^∗^Significant difference.

**Table 6:** *In vivo* hemodynamic function in the GK rat.

Parameter	Age	Control versus GK	Reference
HR	15 and 22 w	344.7 versus 314.1^∗^ [15] and 333.1 versus 296.7^∗^ [22] (bpm)	[34]
	14–16 w	322 versus 328 bpm NSD	[28]
	16 w	NSD	[37]
	16 w	453 versus 454 bpm NSD	[30]
	18 w	369 versus 417 bpm NSD	[31]
	20 w	208 versus 217 bpm NSD	[32]
	20 w	341 versus 360 bpm NSD	[33]
	15 and 22 w	344.7 versus 314.1^∗^ [15] and 333.1 versus 296.7^∗^ [22] (bpm)	[34]
	24 w	370.33 versus 323.00^∗^ (bpm)	[2]
	18 and 30 w	337 versus 350 [18] and 319 versus 328 bpm [30] NSD	[45]
	2, 7, and 15 m	370 versus 316^∗^ [2], 324 versus 264^∗^ [7], and 307 versus 256^∗^ [15] (bpm)	[46]
	3 m	NSD	[58]

SBP	15 and 22 w	122.3 versus 138.4^∗^ [15] and 117.5 versus 135.0^∗^ [22] (mmHg)	[34]
	14–16 w	131 versus 134 mmHg NSD	[28]
	16 w	Higher^∗^	[37]
	16 w	145 versus 123 mmHg NSD	[30]
	18 w	117 versus 121 mmHg NSD	[31]
	20 w	Higher^∗^	[32]
	20 w	144 versus 149 mmHg NSD	[33]
	15 and 22 w	122.3 versus 138.4^∗^ [15] and 117.5 versus 135.0^∗^ [22] (mmHg)	[34]
	3 m	124 versus 152^∗^ (mmHg)	[58]

DBP	15 and 22 w	88.1 versus 95.4^∗^ [15] and 84.0 versus 91.6 [22] (mmHg)	[34]
	16 w	117 versus 89^∗^ (mmHg)	[30]
	15 and 22 w	88.1 versus 95.4^∗^ [15] and 84.0 versus 91.6 mmHg [22]	[34]

MAP	16 w	117 versus 120 mmHg NSD	[35]
	16 w	Higher^∗^	[37]
	16 w	126 versus 100^∗^ (mmHg)	[30]

PLVP	18 and 30 w	106 versus 105 [18] and 112 versus 108 mmHg [30] NSD	[45]

LV +dP/dt	18 and 30 w	6510 versus 5953 [18] and 6846 versus 5840 mmHg/s [30] NSD	[45]
	26 w	NSD	[30]

LV –dP/dt	18 and 30 w	4800 versus 4614 (18) and 5166 versus 5111 mmHg/s [30] NSD	[45]
	26 w	NSD	[30]

LVEDP	18 and 30 w	8 versus 6 [18] and 9 versus 6^∗^ [30] (mmHg)	[45]

LVEDV	20 w	550 versus 713 *μ*l NSD	[32]

LVDV	6 m	411.69 versus 415.53 *μ*l NSD	[51]

LVSV	6 m	108.51 versus 196.01^∗^ (*μ*l)	[51]

EF	14–16 w	80 versus 73^∗^ (%)	[28]
	16 w	NSD	[30]
	20 w	77.9 versus 80.5% NSD	[33]
	26 w	0.74 versus 0.93^∗^ (%)	[44]
	6 m	73.42 versus 52.63^∗^ (%)	[51]

FS	20 w	47 versus 30^∗^ (%)	[32]
	20 w	42.3 versus 45.3% NSD	[33]
	24 w	43.45 versus 38.20% NSD	[2]
	6 m	44.41 versus 28.56^∗^ (%)	[51]
	18 and 30 w	51 versus 55 [18] and 49 versus 51 cm [30] NSD	[45]

CO	20 w	368 versus 321 ml/min NSD	[33]
	6 m	303.7 versus 219.52^∗^ (*μ*l)	[51]

IVCT	24 w	10.98 versus 12.26^∗^ (ms)	[2]

IVRT	14–16 w	25.3 versus 28.3^∗^ (ms)	[28]
	24 w	19.09 versus 24.88 ms	[2]

CBF	15 w	Increased^∗^	[29]
	24 w	4.32 versus 2.46^∗^ (mL/g/min)	[2]

HR: heart rate; SBP: systolic blood pressure; DBP: diastolic blood pressure; MAP: mean arterial pressure; PLVP: peak left ventricular pressure; LV +dP/dt: rate for pressure development in left ventricle; LV –dP/dt: rate for pressure decline in left ventricle; LVEDP: left ventricular end diastolic pressure; LVEDV: left ventricular end diastolic volume; LVDV: left ventricular diastolic volume; LVSV: left ventricular systolic volume; EF: ejection fraction; FS: fractional shortening; CO: cardiac output; IVCT: isovolumic contraction time; IVRT: isovolumic relaxation time; CBF: coronary blood flow; NSD: no significant difference. ^∗^Significant difference.

**Table 7:** Isolated heart hemodynamic function in the GK rat.

Parameter	Age	Control versus GK	Reference
HR	18 w	237 versus 213 bpm NSF	[31]
	5–8 m	251.8 versus 259.5 bpm NSD	[10]
	9–14 m	267 versus 271 bpm NSD	[11]
	18 m	138 versus 115 bpm NSD	[50]

LVP	18 w	44 versus 52 mmHg NSD	[31]
	6 m	Reduced^∗^	[51]

LVDP	5–8 m	126.6 versus 119.8 mmHg NSD	[10]
	6 m	Reduced^∗^	[51]
	16 w	NSD	[35]
	9–14 m	76 versus 63 mmHg NSD	[11]

RPP	16 w	NSD	[35]

EDP	9–14 m	8 versus 10 mmHg NSD	[11]

LV +dP/dt	18 w	1365 versus 1602 mmHg/s NSD	[31]
	5–8 m	3390.6 versus 3169.5 mmHg/s NSD	[10]
	6 m	Reduced^∗^	[51]

LV −dP/dt	18 w	−945 versus −1032 mmHg/s NSD	[31]
	5–8 m	−2669.0 versus −2672.0 mmHg/s NSD	[10]
	6 m	Reduced^∗^	[51]

CF	18 w	7.1 versus 5.8^∗^ (ml/min)	[31]
	5–8 m	10.9 versus 9.8 ml/min/g NSD	[10]
	9–14 m	Reduced^∗^	[11]

CPP	5–8 m	74.2 versus 76.6 mmHg NSD	[10]

HR: heart rate; LVP: left ventricular pressure; LVDP: left ventricular developed pressure; RPP: rate pressure product; EDP: end diastolic pressure; LV +dP/dt: rate for pressure development in left ventricle; LV –dP/dt: rate for pressure decline in left ventricle; CF: coronary flow; CPP: coronary perfusion pressure; NSD: no significant difference. ^∗^Significant difference.

**Table 8:** Myocyte contraction from the GK rat heart.

Parameter	Age	Control versus GK	Reference
MD	8 w	9.11 versus 9.93^∗^ (*μ*m)	[36]
	18 m	9.43 versus 11.34^∗^ (*μ*m)	[40]

SA	16 w	Increased^∗^	[30]
	6 m	Increased^∗^	[51]

CSA	20 w	Increased^∗^	[33]

RCL	8–10 w	NSD	[41]
	5–8 m	NSD	[10]
	10 m	139.48 versus 155.63^∗^ (*μ*m)	[47]
	10-11 m	Increased^∗^	[48]
	17 m	109.7 versus 109.3 *μ*m NSD	[39]
	18 m	139.8 versus 146.4 *μ*m NSD	[50]

CP	14–16 w	Increased^∗^	[28]

TPK	8–10 w	115.03 versus 125.38^∗^ (ms)	[41]
	10 m	119.77 versus 136.15^∗^ (ms)	[47]
	10-11 m	NSD	[48]
	17 m	302.7 versus 337.5^∗^ (ms)	[39]
	18 m	119.9 versus 115.1 ms NSD	[50]

THALF	8–10 w	NSD	[41]
	10 m	NSD	[47]
	10-11 m	NSD	[48]
	18 m	75.2 versus 65.1^∗^ (ms)	[50]
	17 m	231.3 versus 275.4^∗^ (ms)	[39]

AMP	8–10 w	NSD	[41]
	5–8 m	NSD	[10]
	10 m	6.52 versus 7.15% NSD	[47]
	10-11 m	NSD	[48]
	17 m	5.05 versus 6.56^∗^ (%)	[39]
	18 m	6.7 versus 6.5% NSD	[50]

MD: myocyte diameter; SA: surface area; CSA: cross-sectional area; RCL: resting cell length; CP: cell capacitance; TPK: time to peak shortening; THALF: time to half relaxation of shortening; AMP: amplitude of shortening; NSD: no significant difference. ^∗^Significant difference.

**Table 9:** Myocyte calcium from the GK rat heart.

Parameter	Age	Control versus GK	Reference
RCa^2+^	14–16 w	0.97 versus 1.25^∗^ (RU)	[28]
	8–10 w	NSD	[41]
	5–8 m	NSD	[10]
	10 m	NSD	[47]
	10-11 m	NSD	[48]
	17 m	1.32 versus 1.23 RU NSD	[39]
	18 m	1.28 versus 1.31 RU NSD	[50]

TPK	8–10 w	NSD	[41]
	10 m	55.82 versus 66.14^∗^ (ms)	[47]
	10-11 m	NSD	[48]
	17 m	91.7 versus 104.3 ms NSD	[39]
	18 m	64.8 versus 66.6 ms NSD	[50]

THALF	8–10 w	183.46 versus 148.32^∗^ (ms)	[41]
	10 m	NSD	[47]
	10-11 m	NSD	[48]
	17 m	199.1 versus 199.0 ms NSD	[39]
	18 m	136.2 versus 123.1 ms NSD	[50]

AMP	8–10 w	NSD	[41]
	5–8 m	NSD	[10]
	10 m	0.25 versus 0.31 (RU)	[47]
	10-11 m	NSD	[48]
	17 m	0.30 versus 0.23^∗^ (RU)	[39]
	18 m	0.50 versus 0.78^∗^ (RU)	[50]

ICaL amplitude	10-11 m	NSD	[48]

ICaL inactivation	10-11 m	NSD	[48]

ICaL restitution	10-11 m	NSD	[48]

MS Ca^2+^	17 m	31.9 versus 89.2^∗^ (*μ*m/RU)	[39]

RCa^2+^: resting Ca^2+^; TPK: time to peak Ca^2+^ transient; THALF: time to half decay of the Ca^2+^ transient; AMP: amplitude of the Ca^2+^ transient; ICaL: L-type Ca^2+^ current; MSCa^2+^: myofilament sensitivity to Ca^2+^; NSD: no significant difference. ^∗^Significant difference.

## Abbreviations

DM: Diabetes mellitus
CVDs: Cardiovascular diseases
T2DM: Type 2 diabetes mellitus
GK: Goto-Kakizaki
GLUT-2: Glucose transporter
GPDH: Glycerol-3-phosphate dehydrogenase
NZO: New Zealand obese
OLETF: Otsuka Long Evans Tokushima Fat
SDT: Spontaneously diabetic Torri
TGF-*β*1: Transforming growth factor-*β*1
ECM: Extracellular matrix
PKB: Protein kinase B
ECC: Excitation-contraction coupling
SR: Sarcoplasmic reticulum
RyR: Ryanodine receptor
CICR: Calcium-induced calcium release
SERCA2: SR Ca^2+^-ATPase2
NCX: Na^+^/Ca^2+^ exchanger
ENDO: Endocardial
EPI: Epicardial.
